# Disease activity index is associated with subclinical atherosclerosis in childhood-onset systemic lupus erythematosus

**DOI:** 10.1186/s12969-021-00513-5

**Published:** 2021-03-20

**Authors:** Priscila B. S. Medeiros, Roberta G. Salomão, Sara R. Teixeira, Diane M. Rassi, Luciana Rodrigues, Davi C. Aragon, Priscila G. Fassini, Fábio V. Ued, Rita C. Tostes, Jacqueline P. Monteiro, Virgínia P. L. Ferriani, Luciana M. de Carvalho

**Affiliations:** 1 Department of Pediatrics, University of São Paulo, Av. Bandeirantes, 3900, Ribeirão Preto, São Paulo 14049-900 Brazil; 2 Division of Pediatric Rheumatology, University of São Paulo, Ribeirão Preto, São Paulo Brazil; 3 Division of Nutrition and Metabolism, Department of Health Sciences, Ribeirão Preto Medical School, University of São Paulo, Ribeirão Preto, São Paulo Brazil; 4 Department of Medical Imaging, Oncology and Hematology, University of São Paulo, Ribeirão Preto, São Paulo Brazil; 5 Department of Pharmacology, University of São Paulo, Ribeirão Preto, São Paulo Brazil; 6 Department of Internal Medicine, Division of Nutrology, University of São Paulo, Ribeirão Preto, São Paulo Brazil; 7 Clinical Hospital of Ribeirão Preto Medical School, University of São Paulo, Av. Bandeirantes, 3900, Ribeirão Preto, SP 14049-900 Brazil

**Keywords:** Atherosclerosis, Lupus Erythematosus, systemic, childhood, Carotid intima-media thickness, Nutritional assessment

## Abstract

**Background:**

Systemic lupus erythematosus (SLE) is an independent risk factor for cardiovascular events. The present study determined the prevalence of subclinical atherosclerosis in childhood-onset SLE using the carotid intima-media thickness (CIMT) measurement and investigated associations between traditional and nontraditional risk factors for atherosclerosis, such as medications, SLE Disease Activity Index - SLEDAI-2 K and SLICC-ACR damage index and CIMT.

**Methods:**

Cross-sectional prospective study between 2017 and 2018. CIMT was assessed by ultrasonography. Data were collected by chart review, nutritional evaluation and laboratory tests and analyzed by Fisher, Wilcoxon-Mann-Whitney tests, multiple linear and log binomial regression.

**Results:**

Twenty-eight patients (mean age 13.9 years, SD 3) were enrolled. The prevalence of subclinical atherosclerosis was 32% (95% CI 14.8, 49.4). The mean CIMT was 0.43 ± 0.035 mm. The most common traditional risk factors observed were dyslipidemia (82.1%), uncontrolled hypertension (14.2%), obesity (14.3%), and poor diet (78.6%). Uncontrolled hypertension (*p* = 0.04), proteinuria (*p* = 0.02), estimated glomerular filtration rate < 75 ml /min/1.73 m^2^ (*p* = 0.02) and SLEDAI-2 K > 5 (*P* = 0.04) were associated with subclinical atherosclerosis. SLEDAI-2 K > 5 maintained association with CIMT after adjusting for control variables.

**Conclusion:**

Subclinical atherosclerosis is frequently observed in cSLE, mainly in patients with moderate to severe disease activity.

## Background

Early diagnosis and treatment advances have increased the survival of systemic lupus erythematosus (SLE) patients over the last decades. Nonetheless, comorbidities, in particular cardiovascular diseases (CVD), still predispose these patients to higher mortality and morbidity [1].

The presence or increase of traditional Framingham risk factors for CVD is insufficient to explain the high CVD index in SLE patients [2]. CVD in SLE is multifactorial. Factors related to the disease itself, such as inflammation, immune complex-mediated endothelial damage, nephrotic proteinuria and its complications, contribute to the pathogenesis of CVD in SLE patients [3, 4].

Childhood-onset systemic lupus erythematosus (cSLE) is more aggressive and carries higher chances of early sequelae when compared to adult-onset SLE [5].

Therefore, early detection and prevention of traditional (like hypertension, diabetes, smoking, contraceptives use, higher body mass index and waist circumference, and dyslipidemia) and nontraditional risk factors (like longer SLE duration, increased creatinine clearance, active disease and corticosteroids use) for atherosclerosis in cSLE are of utmost importance for hampering development of CVD in these patients [6].

Carotid intima-media thickness (CIMT) assessed by ultrasonography (US) has been used as a surrogate marker of subclinical atherosclerosis in children and young adults with high accuracy [7]. Increments of 0.1 mm of the CIMT, increases the risk for acute myocardial infarction by 10 to 15% and stroke by 13 to 18% [8].

Considering that CIMT, a biomarker for subclinical atherosclerosis, may be associated with traditional and nontraditional risk factors for atherosclerosis, the aims of the study were i) to determine the prevalence of subclinical atherosclerosis assessed by CIMT in patients with cSLE; ii) to investigate associations between CIMT and traditional and nontraditional risk factors for atherosclerosis.

## Methods

### Patients

This cross sectional study was performed between April 2017 and May 2018. cSLE patients followed in the Paediatric Rheumatology Clinic of the Clinical Hospital of Ribeirão Preto Medical School, Brazil, a tertiary referral center, were invited to participate. All patients fulfilled the American College of Rheumatology classification criteria for SLE [9], had disease onset at less than age 18 years, and were recruited up to 21 years of age. Pregnant individuals and patients with infection were excluded.

### Clinical evaluation

Clinical data, such as demographics, lupus-related clinical history, comorbidities, traditional risk factors for atherosclerosis, including hypertension, dyslipidemia, obesity, diabetes and use of contraceptives were retrieved from the patient’s medical records. Current and previous medication treatment - prednisone, intravenous methylprednisolone pulse, hydroxychloroquine sulphate, methotrexate, azathioprine, cyclosporine, mycophenolate and intravenous cyclophosphamide - were also recorded.

The prescribed corticoid was evaluated in total actual prednisone equivalent dose (mg), accumulated dose in grams, and in average dose by kg/day during the patient treatment. The patients were stratified into three groups based on the average dose of corticoid used for disease treatment: low dose, < 0.15 mg/ kg/day; moderate dose, 0.15–0.40 mg/ kg/day; high dose, > 0.40 mg/kg/day. The categorization of the corticosteroid dose was based on the relationship between the weight-adjusted prednisone dose and the CIMT measurement identified in the APPLE study [6].

The Systemic Lupus Erythematosus Disease Activity Index - SLEDAI-2 K were used to assess disease activity and to classify the patients by activity level: 0 no activity, 0–5 mild activity and, greater than 5 moderate to severe [10].

The Systemic Lupus International Collaborating Clinics/American College of Rheumatology Damage Index (SDI) - SLICC/ACR DI were used to assess disease activity and cumulative damage [11].

Physical examination of all patients was performed by the same pediatric rheumatologist (PBSM). Tanner’s pubertal staging was assessed to classify the patients into pre-puberty and puberty (Tanner stage ≥2) since that puberty may be related to cSLE disease activity [12, 13]. Hypertension was defined as systolic and/or diastolic blood pressure  95th percentile for children between 1 and 13 years and ≥ 130/80 mmHg for children ≥13 years [14].

Data on diet, smoking and physical activity were collected during the physical evaluation. Regular physical activity was considered when the patient performed 60 min or more of daily physical activity, with periods of vigorous activity performed at least 3 days per week, for the last 3 months [15].

### Laboratory assessment

Twelve-hour fasting exams included complete blood cell count, complement levels (C3 and C4), anti-dsDNA, homocysteine, high sensitivity C-reactive protein (hsCRP), cyanocobalamin, folate, total cholesterol, triglycerides, high density lipoprotein, low density lipoprotein, glucose levels, urine analysis and spot urine protein creatinine ratio. Cut-off values were determined based on the assay manufacturer’s instructions.

Proteinuria was defined as protein to creatinine ratio > 0.2 in spot urine sample and moderate to severe proteinuria for ratio ≥ 1 [16]. Creatinine clearance (CrCl) was calculated using the Schwartz formula [17].

Plasma levels of interleukins (IL), IL-1alpha and IL-1beta, IL-6, IL-10, IL-17; interferon (IFN) alpha and beta and adhesion molecules, vascular cell adhesion molecule 1 (VCAM-1), intercellular adhesion molecule 1 (ICAM-1) and P-selectin were measured using commercial Kits - Human ELISA kits - R&D Systems Inc.® (Minneapolis, MN, USA).

### Nutritional assessment

The anthropometric measurements were weight, height, body mass index (BMI) [18] and waist circumference (WC) [19], measured according to published methods. Electrical bioimpedance analysis was performed and the percentage of body fat was determined according to McCarthy, 2006 [20].

Food intake was assessed by one 24-h dietary recall (24 h-R) applied to participants on the same day of physical exam and blood sample collection [21]. A picture booklet of food portion size (small, medium and large) was used to convert the food to g or ml [22]. Energy, macronutrients and micronutrients were calculated using the software specially developed to analyze the dietary intake of the Brazilian population (Dietwin® version 1997–2002).

The Healthy Eating Index (HEI) was calculated using data obtained from the 24 h-R. The HEI considered in this study was previously revised for the Brazilian population [23] and subsequently validated for children and adolescents [24]. It consists of an indicator that simultaneously analyzes several nutritional components, based on energy density, evaluating their quality, regardless of the amount of food consumed, represented by a single value that can be classified as: “poor diet”, scores less than 65; “good diet”, scores ≥85; and two intermediate categories, 65–74 and 75–84, “need adaptation in the diet” [24]. The maximum index score is 100.

### Carotid intima-media thickness (CIMT)

**Fig. 1:**
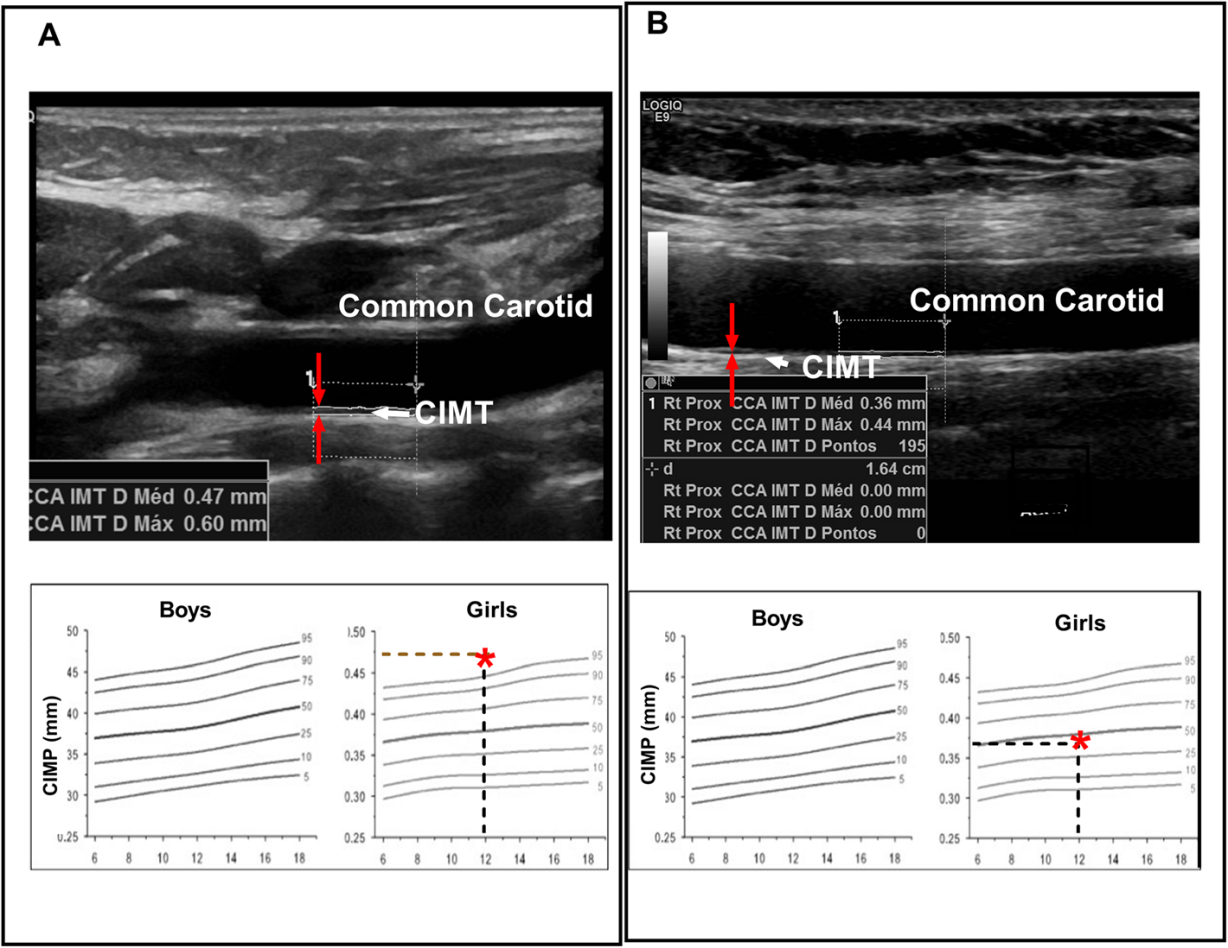
Example of carotid intima-media thickness (CIMT) of the far wall of the common carotid artery. **a** Patient with mean CIMT of 0.47 mm above the 90th percentile for age and sex controls *(Doyon* et al*, Hypertension, 2013)*, which is considered subclinical atherosclerosis. **b** Another patient with a mean CIMT of 0.36 mm within the normal range for age

To determine the prevalence of subclinical atherosclerosis an expert radiologist (SRT) performed US to assess the CIMT using a Logic E9 device (General Electric Company) with high frequency linear transducers (ML6–15 MHz) and following international standardized protocols [7]. US was performed in a controlled room temperature at 23 °C, in the morning (between 7:30 am and 9:00 am), with the patient resting comfortably for 15 min prior to acquiring data in the supine position. The patient’s neck was slightly extended and the head turned 45° towards the side opposite of that being examined. CIMT was automatically measured on longitudinal B-mode images using the Auto IMT software. After identifying the carotid bifurcation, CIMT was measured from three arterial segments three times each, as follows: the common carotid artery (CCA) from 10 mm proximal to the tip of the flow divider (TFD), the carotid bifurcation (from the TFD to 10 mm proximal to the TFD) and the proximal 10 mm of the internal carotid artery (ICA). The average of the three measures of each segment was used to compose data. CIMT measurements were also compared to reference values of a population study for children under 18 years old [25, 26] and for teenagers between 18 and 21 years old [27, 28]. CIMT greater than or equal to the 90th percentile for age and sex was considered subclinical atherosclerosis [29] (Fig. 1).

### Statistical analysis

The characteristics of the study sample were summarized using descriptive statistics with ordinal data presented as percentages and continuous data as means, standard deviations [SD] or medians and interquartile ranges, as appropriate. The study sample was divided into two groups, with and without subclinical atherosclerosis, according to the results of the CIMT measurements. Fisher’s exact test and Wilcoxon-Mann Whitney test were used to investigate associations between categorical and continuous variables with groups, respectively. Fitted log-binomial regression models were used to estimate prevalence ratios and their 95% confidence intervals. Linear regression models were adjusted to associate potential risk factors with CIMT. Statistical and clinical criteria were considered for selecting variables for multiple modeling.

Multiple correlation between continuous variables and CIMT measurements was assessed with Spearman’s rank order correlation coefficient. Both the absolute values and normalized percentiles of the CIMT measurements were analyzed. For statistical purposes, the CIMT values of the right and left carotids were averaged.

The SLEDAI-2 K was used as a continuous variable or as a dichotomous variable: SLEDAI-2 k > 5 score (yes or no), for considering SLEDAI values greater than 5 moderate to severe disease activity. The SLICC/ACR DI scores were included in the statistical analysis as a dichotomous variable with subjects grouped as score of 0 (no damage) or score ≥ 1 (damage present) [30].

The analysis was performed using the SAS 9.2 software (The SAS system for Windows, release 9.2. SAS Inst., Cary, NC. 2011 and R 3.5.1 software). The Stata software (version 10.0) was used for calculating the IQD-R. *P* values < 0.05, were considered to be statistically significant.

## Results

Twenty-eight patients with cSLE were enrolled in the study.

The point prevalence coefficient of subclinical atherosclerosis was 32.14% (CI 95%: 14.8; 49.4) in the evaluated cSLE patients.

Of the 28 enrolled patients, 78.6% were female, 60.7% non-caucasian, the mean age at inclusion was 13.9 years (range 6.4–19.5 years) and 96.4% had Tanner stage ≥2.

The mean disease duration was 30 months (range 1–110 months). The most frequent manifestations were nephritis (89.2%) – 42.8% of the patients had the International Society of Nephrology and the Renal Pathology Society (ISN/RPS) nephritis classification class IV and 14.2% class V, three patients with estimate creatinine clearance (CrCl) < 75 ml/min/1.73m^2^ and 28.6% with moderate or severe proteinuria; hematological alteration (78.6%) and arthritis (64.2%). Antiphospholipid and anti-ds-DNA antibodies were positive in 50 and 75% of the patients, respectively.

Eighty nine percent of patients had active disease, 60.7% moderate to severe disease activity, based on SLEDAI score > 5, and 25% had damage as measured by the SLICC > 0.

All patients were on hydroxychloroquine use and 92.8% on corticoid use. The mean current total corticoid dose was 9 mg (0–40 mg), median 5 mg/day and accumulated dose used during patient treatment was 19 g (range 0–60.4), median 13 g. In relation to the average dose of corticoid by kg/day during the treatment, 2 patients (7%) used < 0.15 mg/kg/day; 14 (50%) used 0.15–0.40 mg/kg/day and 10 (36%) used doses > 0.40 mg/kg/day.

Cyclophosphamide was the most used immunosuppressant (53%), followed by azathioprine (32%) and mycophenolate (28.6%). Thirty five percent of the patients were on antihypertensive drugs and 17.8% on acetylsalicylic acid (0.5 mg/kg/day).

The traditional risk factors were dyslipidemia (82.1%) - low levels of high-density lipoprotein (57.1%), hypertriglyceridemia (35.7%), uncontrolled hypertension (14.2%) and obesity (14.3%). One patient had diabetes and three of the female patients, used oral contraceptive. No patient was a smoker. No patient had regular practice of physical activity.

Anthropometric data showed that 64.3% of the patients had adequate height for sex and age. Only 4 patients (14.3%) were classified as obese by BMI, and bioimpedance analysis detected excess body fat in 7 (25%) patients. However, 13 (46.4%) patients exhibited high waist circumference, a factor related to a higher risk of cardiovascular disease. No patient presented adequate food intake based on the Healthy Eating Index (HEI > 84). “Poor diet” was reported by 78.6% of the individuals (HEI < 65).

The mean CIMT was 0.43 ± 0.035 mm. Nine patients (32.14%), of whom 8 were female, had the diagnosis of subclinical atherosclerosis based on a CIMT of greater than or equal to the 90th percentile.

The frequency of boys under 18 years with CIMT means in the 75-90th percentile and 90-95th percentile were 66.6 and 16.7%, respectively and one boy over 18 years (16.7%) was in the 10-90th CIMT percentile. In girls under 18 years, the frequency of CIMT means in 50-75th, 75-90th, 90-95th and above 95th percentile were 45.4, 13.6, 9.1 and 22.7%, respectively; one girl over 18 years was in the 10-90th (4.5%) and one above 90th (4.5%) CIMT percentile (Fig. 1).

For univariable relationships analysis between clinical risk factors and subclinical atherosclerosis, the population was categorized into two groups, with or without subclinical atherosclerosis. The median of CIMT was also compared between risk factors (Tables 1 and 2).

**Table 1:** Univariate relationships between clinical risk factors and subclinical atherosclerosis and CIMT in juvenile systemic lupus study participants

Variable (n)	Subclinical Atherosclerosis n (%) or mean [SD]		***P*** value*	CIMT – mm mean [SD]		***P*** value*
	Yes	No		Opposite Dichotomic Control		
Female (22)	8 (36.4)	14 (63.6)	0.68	0.42 [0.04]	0.46 [0.05]	0.08
Caucasian (11)	5 (45.4)	6 (54.5)	0.41	0.43 [0.04]	0.44 [0.04]	0.69
Age	13 [3.3]	14.4 [2.8]	0.29			
Disease duration in months	39 [34.2]	25.7 [20.4]	0.34			
**Clinical features**						
Nephritis (25)	8 (32)	17 (68)	1	0.43 [0.04]	0.42 [0.02]	0.74
Class IV and/or V (16)	7 (43.7)	9 (56.2)	0.12	0.44 [0.04]	0.41 [0.02]	0.052
Neuropsychiatric symptoms (8)	3 (37.5)	5 (62.5)	1	0.43 [0.04]	0.43 [0.04]	1
SLEDAI-2 k > 5 (17)	9 (52.9)	8 (47)	**0.04**	0.44 [0.05]	0.41 [0.02]	**0.04**
SLEDAI-2 k	10.9 [5,7]	6.4 [5.6]	0.05			
SLICC/ACR DI > 0 (7)	2 (28)	5 (71)	1	0.43 [0.03]	0.43 [0.05]	0.83
Uncontrolled hypertension	3 (75)	1(25)	0.08	0.46 [0.02]	0.43 [0.04]	**0.04**
Menarche (15)	4 (26.7)	11 (73.3)	0.34	0.42 [0.03]	0.43 [0.05]	0.68
Tanner stage ≥2 (27)	8 (29.6)	19 (70.3)	0.32	0,43 [0,04]	0,43	
Short stature (5)	2 (40)	3 (60)	1	0.46 [0.03]	0.43 [0.04]	**0.04**
Cumulative dose prednisone						
< 0.15 mg/kg/day (2)	0	2 (100)	1	0.39 [0.02)	0.43 [0.04]	0.12
0.15–0.40 mg/kg/day (14)	5 (35.7)	9 (64.3)	1	0.44 [0.04]	0.42 [0.04]	0.38
> 0.40 mg/kg/day (10)	3 (30)	7 (70)	1	0.42 [0.03]	0.44 [0.05]	0.74
Actual prednisone dose (mg/ day)	0.4 [0.3]	0.5 [1]	0.64			
Cumulative dose prednisone	22.6 [18.1]	16.8 [14.8]	0.46			
Cyclosphosphamide (15)	5 (33)	10 (67)	1	0.44 [0.04]	0.42 [0.04]	0.14
Azathioprine (9)	3 (33)	6 (67)	1	0.43 [0.03]	0.43 [0.05]	0.85
Mycophenolate (8)	2 (25)	6 (75)	1	0.44 [0,05]	0.43 [0,04]	0.44
Oral contraceptive use	1 (33.3)	2 (66.7)	1	0.44 [0.04]	0.42 [0.04]	0.42
**Nutritional assessment**						
Excess abdominal fat (WC) (13)	2 (15.4)	11 (84.6)	0.11	0.42 [0.04]	0.44 [0.04]	0.22
Overweight (BMI) (5)	1 (20)	4 (80)	1	0.43 [0.07]	0.43 [0.04]	0.46
Obesity (BMI) (4)	0	4 (100)	0.27	0.43 [0.02]	0.43 [0.04]	0.74
**Bioimpedance**						
Excess body fat (7)	3 (42.9)	4 (57.1)	0.33	0.42 [0.02]	0.43 [0.04]	0.65
**Health eating index**						
Poor diet – HEI < 65 (22)	8 (36.4)	14 (63.6)	0.63	0.43 [0.03]	0.44 [0.06]	0.89
Diet in need to adequate (6)	1 (16.7)	5 (83.3)	0.62	0.44 [0.06]	0.43 [0.03]	0.89

*Fisher and Wilcoxon-Mann-Whitney tests. *m* media, *mm* millimeters, *SD* standard deviation, *SLEDAI 2 K* Systemic Lupus Erythematosus Disease Activity Index, *SLICC/ACR DI* Systemic Lupus International Collaborating Clinics/American College of Rheumatology Damage Index, *CIMT* carotid intima-media thickness, *WC* waist circumference, *BMI* body mass index, *HEI* healthy eating index

**Table 2:** Univariate relationships between laboratory evaluation, subclinical atherosclerosis and CIMT in juvenile systemic lupus study participants

Variable (n)	Subclinical Atherosclerosis n (%) or mean [SD]		***P*** value* #	CIMT – mm mean [SD]		***P*** value #
	Yes (9)	No (19)		Opposite dichotomic Control		
Anemia	4 (40)	6 (60)	0.67	0.44[0.03]	0.43[0.05]	0.22
Leukopenia	2 (66.7)	1 (33.3)	0.23	0.43[0.03]	0.43[0.04]	0.58
Lymphopenia	3 (27.3)	8 (72.7)	1	0.42[0.42]	0.44[0.05]	0.40
Hematuria	5 (45.4)	6 (54.5)	0.40	0.44[0.05]	0.43[0.04]	0.29
Leukocyturia	5 (50)	5 (50)	0.21	0.44[0.05]	0.43[0.04]	0.77
Urinary casts	1 (50)	1 (50)	1	0.41[0.03]	0.43[0.04]	0.45
Proteinuria**	7 (58)	5 (41.7)	**0.02**	0.45[0.04]	0.42[0.04]	**0.02**
Proteinuria moderate to severe	5 (62.5)	3 (37.5)	0.06	0.44[0.03]	0.43[0.04]	0.28
Estimate CrCl < 75 ml/min/1.73m^2^	3 (100)	0	**0.02**	0.47[0.02]	**0.43[0.04]**	**0.04**
Estimate CrCl	125.5 [58.9]	162.8 [42.9]	0.14			
Anti-dsDNAn	7 (33)	14 (66,7)	1	0.43[0.04]	0.43[0.05]	0.79
Antiphospholipid positive	7 (50)	7 (50)	0.10	0.42[0.04]	0.44[0.05]	0.68
Low complement C3	5 (38)	8 (61)	0.69	0.43[0.05]	0.43[0.04]	0.75
C4	4 (36.4)	7(63.6)	1	0.43[0.04]	0.43[0.04]	0.74
Dyslipidemia	8 (34.8)	15 (65.2)	1	0.43[0.04]	0.43[0.06]	0.95
Triglycerides (≥130 mg/dL)	5(50)	5(50)	0.2	0.43[0.04]	0.43[0.05]	0.94
Triglycerides	189.5 [139.8]	118.3 [82.4]	0.07			
Total Cholesterol (> 170 mg/dL)	6 (60)	4 (40)	**0.03**	0.44[0.03]	0.43[0.05]	0.24
Total Cholesterol mean [SD]	196.1 [52.5]	148.2 [33.9]	**< 0.01**			
LDL	2 (50)	2 (50)	0.57	0.44[0.05]	0.43[0.04]	0.54
LDL means [SD]	84.9 [22.1]	112.6 [47.9]	0.14			
HDL (< 45 mg/dL)	5 (31.2)	11 (68.7)	1	0.43[0.04]	0.44[0.04]	0.66
HDL means [SD]	43 [8]	37.3 [11.9]	0.20			
High hsCRP	1 (14.3)	6 (85.7)	0.37	0.42[0.03]	0.44[0.04]	0.58
Hyperhomocysteinemia	2 (67)	1 (33)	0.23	0.45[0.02]	0.43[0.04]	0.24
Homocystein mean [SD]	8.4 [3.2]	15 [14]	0.18			
Low cyanocobalamin	0	1(100)	1			
Cyanocobalamin mean [SD]	433.7 [221.5]	411.5 [201]	0.81			
Low folate	0	1(100)	1			
Folate mean [SD]	9.4 ± 3.7	10.7 ± 3.8	0.5			
IL-1 alpha	1 (33.3)	2(66.7)	1	0.46[0.08]	0.43[0.04]	0.75
IL-17 (2)	0	2 (100)	1	0.42[0.01]	0.44[0.05]	0.39
INF-gamma (4)	1 (25)	3 (75)	1	0.45[0.06]	0.43[0.04]	0.84
IL-10 (7)	2(28.6)	5 (71.4)	1	0.42[0.02]	0.45[0.05]	0.46
IL-1 beta (4)	0	4(100)	0.25	0.41[0.02]	0.44[0.05]	0.24
TNF- alpha (4)	0	4 (100)	0.25	0.41[0.02]	0.44[0.05]	0.24
IL-6 mean [SD]	8.6 [10.5]	14.4 [22.8]	0.42			
ICAM-1 mean [SD]	302.9 [81.4]	231.8 [100.9]	0.12			
VCAM-1 mean [SD]	728.2 [626]	418.9 [237.4]	0.42			
P-selectina mean [SD]	52.0 [21.3]	191.9 [288.5]	0.11			

*Fisher and ^#^Wilcoxon-Mann-Whitney tests. ** protein to creatinine ratio ≥ 0.2. *LDL* low density lipoprotein, *HDL* high density lipoprotein, *hsCRP* high sensitivity c-reactive, protein, *IL* interleukins, *IFN* interferon, (IFN), *VCAM-1* vascular cell adhesion molecule-1, *ICAM-1* intercellular adhesion molecule-1 and P-selectin high sensitivity c-reactive, protein (hsCRP), *CIMT* carotid intima-media thickness, *SD* standard deviation, *CrCl* creatinine clearance, mm: millimeters

In 19/28 (67.8%) patients, no significant correlations were found between the plasma levels of interleukins (IL), IL-1alpha, IL-1beta, IL-6, IL-10, IL-17; interferon (IFN) alpha and gamma and adhesion molecules, vascular cell adhesion molecule (VCAM-1), intercellular adhesion molecule-1 (ICAM-1) or P-selectin and subclinical atherosclerosis.

Parameters more frequently observed in patients with subclinical atherosclerosis were: SLEDAI-2 k > 5 (*p* = 0.04), estimated CrCl < 75 ml/min/1.73 m^2^ (*p* = 0.02), protein to creatinine ratio > 0.2 (*p* = 0.02) and higher total cholesterol (*p* = 0.03). Protein to creatinine ratio ≥ 1, moderate to severe proteinuria (*p* = 0.06) and the average of SLEDAI-2 K absolute value (*p* = 0.05) had approached significance.

**Fig. 2:**
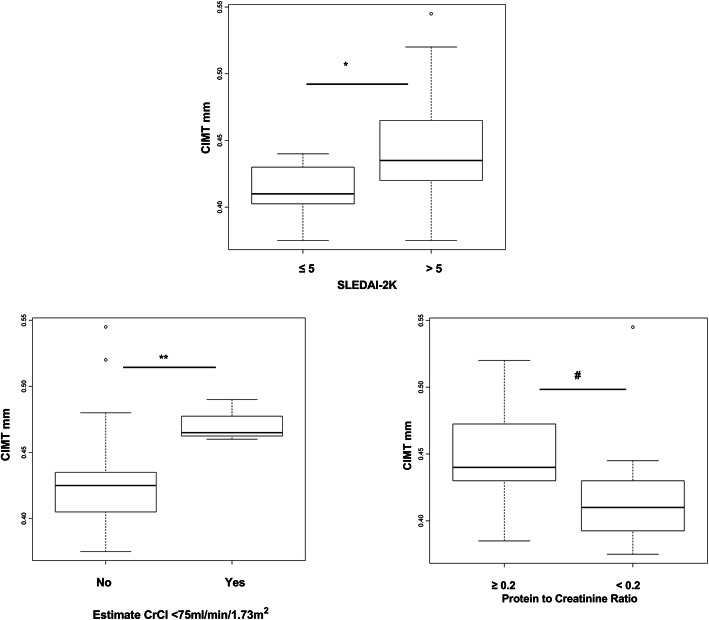
Carotid intima-media thickness (CIMT) measures in mm in cSLE patients according to SLEDAI-2 k score (≤5 ou > 5), Estimate CrCl < 75 ml/min/1,73 m^2^, protein to creatinine ratio in cSLE patients. Value distribution is shown by boxplot (Whiskers 5–95 percentile). Data were analyzed with Mann-Whitney non-parametric test (*P = 0.04 ** P = 0.04 and ^*#*^*P = 0.02)*

When the media of CIMT in mm was compared between non-traditional atherosclerosis risk factors related to SLE, the correlation remained in relation to the SLEDAI > 5 (*p* = 0.04), estimated creatinine clearance < 75 ml/min/1.73 m2 (*p* = 0.04), protein to creatinine ratio above 0.2 (*p* = 0.02) (Fig. 2) and were also significant the presence of uncontrolled hypertension (*p* = 0.04) and short stature (*p* = 0.04).

There was no perfect or strong Spearman correlation coefficient (rho) value between carotid intima-media thickness and studied variables. Cumulative prednisone dose (rho = 0.52; *p* < 0.01) and disease duration in months (rho = 0.52; *p* < 0.01) had moderate positive correlation with CIMT.

Multivariable adjusted association between clinical risk factors and CIMT in mm are reported in Table 3. Variables with significant correlation – SLEDAI-2 k > 5, estimated creatinine clearance < 75 ml/min/1.73 m^2^, protein-creatinine ratio > 0.2 and moderate to severe proteinuria were adjusted for cumulative prednisone dose used and disease duration in months, total cholesterol, triglycerides, high-density lipoprotein, low-density lipoprotein, presence of uncontrolled hypertension and short stature. Only the SLEDAI-2 k > 5 remained significant (Table 3).

**Table 3:** Multiple adjusted relationships between clinical risk factors and CIMT

Carotid intima-media thickness (CIMT) in mm									
		Simple model				Multiple model			
Mo	Variable	MD	*p*-value	95%CI		MD	*p*-value	95%CI	
				LL	UL			LL	UL
1	SLEDAI 2 K > 5	−0.033	**0.03**	−0.064	−0.0029	−0.026	**0.06**	−0.054	0.001
2	SLEDAI 2 K > 5	−0.033	**0.03**	−0.064	−0.0029	−0.038	**0.03**	−0.073	−0.0029
3	SLEDAI 2 K > 5	− 0.033	**0.03**	− 0.064	− 0.0029	− 0.044	**0.02**	− 0.081	− 0.0065
4	Proteinuria	0.032	**0.04**	0.0017	0.06	0.017	0.24	−0.012	0.047
5	CrCl^a^	−0.045	0.07	−0.094	0.004	−0.023	0.41	−0.082	0.034
6	CrCl^a^	−0.045	0.07	−0,094	0.004	0.012	0.87	−0.14	0.16
7	Prot MS	0.011	0.52	−0,024	0.047	0.005	0.84	−0.045	0.055
8	Prot MS	0.011	0.52	−0,024	0.047	0.006	0.7	−0.026	0.039

*mm* millimeters, *CI* confidence interval, *SLEDAI 2 K* Systemic Lupus Erythematosus Disease Activity Index. ^a^CrCl - estimated creatinine clearance < 75 ml / min / 1.73 m2, Proteinuria- protein-creatinine ratio above 0.2; Prot MS -moderate to severe proteinuria; *LL* lower limit, *UL* upper limit, *CI* confidence interval, *MD* difference between the means

Models 1, 4, 5, 8 adjusted by cumulative prednisone dose used and disease duration in months

Model 2 adjusted by total cholesterol, triglycerides, high-density lipoprotein, low-density lipoprotein, low-density lipoprotein

Models 3, 6, 7 adjusted by total cholesterol, triglycerides, high-density lipoprotein, low-density lipoprotein, low-density lipoprotein, presence of uncontrolled hypertension and short stature

## Discussion

The present study shows a prevalence of 32% of subclinical atherosclerosis in childhood-onset systemic lupus erythematosus. Similar data were reported in a five-year follow-up prospective study, with 32% of adult SLE patients displaying evidence of carotid atherosclerosis, compared to 4% in healthy controls [31]. To our knowledge, no other study has yet assessed the prevalence of subclinical atherosclerosis in cSLE based on percentiles of CIMT normality and few studies have assessed the atherosclerosis risk factors in cSLE patients [4, 6, 32, 33].

Atherosclerosis begins in childhood. The PDAY study (Pathobiological Determinants of Atherosclerosis in Youth), which collected data from over 3000 autopsies in 10 to 15 years-old subjects who died from trauma, reported that nearly 100% had atherosclerotic lesions in aorta and 50% in coronary arteries [34]. In particular, pediatric patients with chronic inflammatory disease and chronic kidney disease are at high risk to manifest cardiovascular diseases before the age of 30 years [35]. Thus, cSLE patients need special attention in the surveillance for atherosclerosis and it is important to evaluate the arterial health status of these children and adolescents to prevent atherosclerosis progression.

In our study, traditional risk factors, including dyslipidemia, uncontrolled hypertension, obesity, diabetes and contraceptive use, were frequently observed in cSLE and no patient had adequate food intake. Only one patient had the recommended regular physical activity level [15]. In a previous study, Salomão et al. showed that Brazilian cSLE patients have worse nutritional status based on lipid and proteomic profiles, homocysteine and folate levels and higher BMI and WC when compared to healthy controls [36]. The same was observed in another Brazilian study where adolescent lupus patients exhibited higher levels of total cholesterol, VLDL, triglycerides and homocysteine compared to healthy controls [37]. Abad et al. evaluated the impact of nutritional intervention in these patients. The nutritional intervention in a group of adolescents with lupus for less than 9 months decreased their energy consumption and intake of macronutrients such as carbohydrates, total fat, and saturated fat and had a protective effect against the increase in fat mass [38]. Therefore, the control of traditional risk factors, mainly with nutritional guidance and incentive to physical activity, is the first step towards the prevention of cardiovascular diseases.

Endothelial dysfunction, assessed by arterial compliance and distensibility, and arterial structure deterioration, assessed by CIMT, are early events in the development of cardiovascular disease [7]. Controversial results have been reported in relation to endothelial function analysis (brachial artery flow-mediated dilation) in childhood lupus. Some studies showed no differences on endothelial function between the control and lupus subjects [37, 39], whereas others, mainly studies in adults [32, 40] have shown decreased flow-mediated dilation in lupus patients compared to control subjects. The CIMT exam has limitations represented by being difficult to perform, especially in children, due to the arterial size, to obtain precise measurements and the need to perform measurements at the same time during the cardiac cycle [7].

Assessment of CIMT with high-resolution B-mode ultrasonography has emerged as one of the most powerful tools for the evaluation of subclinical atherosclerosis [7].

In the present study, both the absolute values and normality percentiles of the ultrasound measurements were analyzed, to minimize the limitation of not having a control group and normal reference values for the Brazilian population. The two analysis obtained similar results regarding the correlation between CIMT in mm or subclinical atherosclerosis by percentiles [25–28]: in the univariate analysis the associations with SLEDAI-2 k > 5, estimated CrCl < 75 ml / min / 1.73 m2, protein to creatinine ratio > 0.2 were statistically significant. The presence of uncontrolled hypertension and short stature were associated only with the media of CIMT in mm and the cholesterol with subclinical atherosclerosis group by percentiles. Only the SLEDAI-2 k > 5 maintained significant correlation after the adjusted models.

In the Atherosclerosis Prevention in Pediatric Lupus Erythematosus (APPLE) study, both traditional (increasing age, higher BMI, male sex and higher low-density lipoprotein) and nontraditional risk factors (longer SLE duration, increased creatinine clearance, proteinuria, azathioprine use and prednisone doses) were associated with increased CIMT in 221 lupus patients under 21 years. Vitamin D deficiency was independently associated with elevated hsCRP, a marker of inflammation, which predicts cardiovascular disease risk [6]. In our study, in univariate analysis, only cholesterol levels had a significant association with subclinical atherosclerosis with respect to traditional risk factors and only one patient exhibited vitamin D deficiency.

Although the exact mechanism of atherosclerosis in lupus is not clearly defined, there is evidence that an imbalance between endothelial damage and atheroprotective mechanism seems to be a central event. Insults lead to endothelial damage, and several cytokines and adhesion molecules are involved in this process [41–45].

Neither increased hsCRP nor plasma levels of the cytokines, most frequently correlated with atherosclerosis in previous studies in lupus, including interleukins (IL), IL-1alpha, IL-1beta, IL-6, IL-10, IL-17; interferon (IFN) alpha and gamma, adhesion molecules, vascular cell adhesion molecule (VCAM-1), intercellular adhesion molecule-1 (ICAM-1) or P-selectin, exhibited significant associations with subclinical atherosclerosis. The small sample size may explain the apparently contradictory results in relation to cytokines in our study.

However, moderate to severe disease activity score was an independent risk factor, suggesting that active inflammation may play a role in subclinical atherosclerosis progression. It is possible that other markers of inflammation may be involved, since atherosclerosis is a chronic multifactorial inflammatory disease [46, 47].

Unlike the APPLE study, our study shows no association between CIMT and use of immunosuppressive drugs, such as azathioprine and prednisone, including current and cumulative doses. Since all patients were using hydroxychloroquine, it was not possible to assess a beneficial effect of the drug on serum lipids, as in previous studies [48].

Regarding disease activity, Baragetti et al., considering SLEDAI changes in a longitudinal study, showed that most patients who develop carotid atherosclerosis are characterized by persistent or worsened disease activity during the five-year observation period, independently of traditional risk factors. In addition, specific T cells subsets such CD4+ CCR5 + T cells were independently associated with development of carotid atherosclerosis in SLE patient in this study [31]. Su-Angka et al. reported no significant differences in CIMT between 102 patients with cSLE disease activity and the control group (inactive lupus) [32]. The differences between our study and Su-Angka’s report may be related to the age of patients, since they included younger patients, mean 12 (10.8–15.6) years old in their study in comparison to ours, 13.9 (6.4–19.5) years old. In addition, their report excluded patients with other atherosclerosis risk factors, such as diabetes mellitus and family history of hypercholesterolemia.

The renal involvement, with the highest scores in the disease activity index, has been associated with CIMT. Falashi et al. evaluated twenty-six cSLE patients and reported that patients with nephrotic-range proteinuria have significantly higher CIMT than those without proteinuria [4]. History of lupus nephritis and hypertension were correlated with CIMT in a seven-year surveillance in another SLE study that showed no difference in progression of subclinical atherosclerosis between patients with mild lupus and control subjects [49]. On the other hand, Sharma et al. reported no significant differences in CIMT between 102 SLE patients with and without nephritis, although the nephritis group exhibited a higher SLEDAI score, more persistent inflammation and, consequently, higher risk to developed arterial injury leading to early end-organ damage [50]. In our study estimated CrCl < 75 ml/min/1.73 m^2^ and moderate to severe range proteinuria were associated with CIMT, although no significant association was found after the adjusted models.

Huang et al., in a six-year period longitudinal study, reported that lymphopenia at diagnosis and higher baseline levels of serum creatinine and C-reactive protein are positively associated with progression of CIMT, but only lymphopenia is consistently associated with progression of CIMT in multivariable analysis [33]. Our study showed no specific association with hematological changes and CIMT. However, despite the different results among the studies, they invariably show associations between clinical and laboratory findings and disease activity. Thus, adequate control of disease activity, in addition to monitoring traditional risk factors, is essential to prevent atherosclerosis in cSLE.

Despite great diversity in the studied population and the ethnic origin of cSLE patients, it is agreed that lupus patients are at high risk of developing atherosclerosis, regardless of traditional risk factors.

Longitudinal studies with long time follow up and standardization of validated measurement procedures for the studied population are necessary. Until now, most studies, including ours, correlate active lupus and/or factors related to disease activity with a higher incidence of subclinical atherosclerosis or rapid progression to atherosclerosis.

The CIMT is a non-invasive, reproducible, low cost, and high accuracy tools for the evaluation of subclinical atherosclerosis. But, until now, no standard recommendation is available for monitoring patients for the progression of atherosclerosis with this method in clinical practice, including periodic assessment time [6]. However, international clinical trials were able to demonstrate a significant decrease in CIMT within 1 year with diet and exercise intervention [51] and 2 years after statin therapy intervention [52]. Thus, 1 year is already sufficient for changes in CIMT values. Although longitudinal studies, with a long time follow up and standardization of validated measurement procedures are necessary, we recommend checking the risk factors for atherosclerosis of cSLE patients, including disease activity, at every medical visit, and assessment of the CIMT at least every 2 years.

The early institution of measures to prevent atherosclerosis progression, decreasing long-term cardiovascular complications, could provide a better quality of life and longer survival to our patients.

Our study has limitations that include a small sample size, cross-sectional design and absence of a control group. However, to our knowledge, this is the first study that uses the normative percentile values for CIMT in children and adolescents with lupus to categorize them into two groups, with and without subclinical atherosclerosis. Our data clearly show an adequate and strong association between the mean CIMT in mm, a marker of subclinical atherosclerosis, and disease activity in cSLE.

## Conclusions

Subclinical atherosclerosis is frequently observed in cSLE, mainly in patients with moderate to severe disease activity. Our findings highlight the importance of management of traditional risk factors and adequate control of disease activity to prevent atherosclerosis and consequently cardiovascular diseases.

## Data Availability

The datasets generated and analyzed for the present study are available from the corresponding author on reasonable request.

## Abbreviations

SLE: Systemic lupus erythematosus
CVD: Cardiovascular diseases
cSLE: Childhood-onset systemic lupus erythematosus
CIMT: Carotid intima-media thickness
US: Ultrasonography
SLEDAI-2 K: Systemic Lupus Erythematosus Disease Activity Index 2000
SLICC/ACR DI: Systemic Lupus International Collaborating Clinics/American College of Rheumatology Damage Index (SDI)
anti-dsDNA: Double stranded DNA native antibody
hsCRP: High sensitivity C-reactive
CrCl: Creatinine clearance
IL: Interleukins
IFN: Interferon (IFN)
VCAM-1: Vascular cell adhesion molecule 1
ICAM-1: Intercellular adhesion molecule 1
WC: Waist circumference
HEI: Healthy Eating Index
CCA: Common carotid artery
TFD: Tip of the flow divider
ICA: Internal carotid artery.

## References

[1] Bertsias GK, Salmon JE, Boumpas DT (2010). Therapeutic opportunities in systemic lupus erythematosus: state of the art and prospects for the new decade. Ann Rheum Dis.

[2] Jafri K, Ogdie A, Qasim A, Patterson SL, Gianfrancesco M, Izadi Z (2018). Discordance of the Framingham cardiovascular risk score and the 2013 American College of Cardiology/American Heart Association risk score in systemic lupus erythematosus and rheumatoid arthritis. Clin Rheumatol.

[3] Ahmad Y, Bruce IN (2004). Subclinical atherosclerosis in systemic lupus erythematosus. J Rheumatol.

[4] Falaschi F, Ravelli A, Martignoni A, Migliavacca D, Sartori M, Pistorio A (2000). Nephrotic-range proteinuria, the major risk factor for early atherosclerosis in juvenile-onset systemic lupus erythematosus. Arthritis Rheum.

[5] Barsalou J, Levy DM, Silverman ED (2013). An update on childhood-onset systemic lupus erythematosus. Curr Opin Rheumatol.

[6] Schanberg LE, Sandborg C, Barnhart HX, Ardoin SP, Yow E, Evans GW (2009). Premature atherosclerosis in pediatric systemic lupus erythematosus: risk factors for increased carotid intima-media thickness in the atherosclerosis prevention in pediatric lupus erythematosus cohort. Arthritis Rheum.

[7] Urbina EM, Williams RV, Alpert BS, Collins RT, Daniels SR, Hayman L (2009). Noninvasive assessment of subclinical atherosclerosis in children and adolescents: recommendations for standard assessment for clinical research: a scientific statement from the American Heart Association. Hypertension..

[8] Lorenz MW, Markus HS, Bots ML, Rosvall M, Sitzer M (2007). Prediction of clinical cardiovascular events with carotid intima-media thickness: a systematic review and meta-analysis. Circulation..

[9] Petri M, Magder L (2004). Classification criteria for systemic lupus erythematosus: a review. Lupus..

[10] Gladman DD, Ibanez D, Urowitz MB (2002). Systemic lupus erythematosus disease activity index 2000. J Rheumatol.

[11] Gladman D, Ginzler E, Goldsmith C, Fortin P, Liang M, Urowitz M (1996). The development and initial validation of the systemic lupus international collaborating clinics/American College of Rheumatology damage index for systemic lupus erythematosus. Arthritis Rheum.

[12] Tanner JM (1953). Growth of the human at the time of adolescence. Lect Sci Basis Med.

[13] O’Neil K, Brunner H, Zeft A, Stevens A, Li S, Wright T (2017). Effects of Puberty on Systemic Lupus Erythematosus: Results of a multi-center prospective longitudinal observational study in children entering puberty with SLE [abstract]. Arthritis Rheum.

[14] Flynn JT, Kaelber DC, Baker-Smith CM, Blowey D, Carroll AE, Daniels SR, et al. Clinical Practice Guideline for Screening and Management of High Blood Pressure in Children and Adolescents. Pediatrics. 2017;140(3):e20171904. 10.1542/peds.2017-1904. 10.1542/peds.2017-1904 28827377

[15] How much physical activity do children need? Centers for Disease Control and Prevention Web. http://www.cdc.gov/physicalactivity/everyone/guidelines/children.html.

[16] Ginsberg JM, Chang BS, Matarese RA, Garella S (1983). Use of single voided urine samples to estimate quantitative proteinuria. N Engl J Med.

[17] Schwartz GJ, Haycock GB, Edelmann CM, Spitzer A (1976). A simple estimate of glomerular filtration rate in children derived from body length and plasma creatinine. Pediatrics..

[18] Heymsfield SB, Tighe A, Wang ZM, Shils ME, Olson JA, Shine M (1994). Nutricional assessment by anthopometric and biochemical methods. Modern nutrition in health and disease: Malvem: Lea Febiger.

[19] Fernandez JR, Redden DT, Pietrobelli A, Allison DB (2004). Waist circumference percentiles in nationally representative samples of African-American, European-American, and Mexican-American children and adolescents. J Pediatr.

[20] McCarthy HD, Cole TJ, Fry T, Jebb SA, Prentice AM (2006). Body fat reference curves for children. Int J Obes.

[21] Thompson FE, Byers T (1994). Dietary assessment resource manual. J Nutr.

[22] Monteiro JP, Pfrimer K, Tremeschin MH, Molina MC, Chiarello P, Vannucchi H (2007). Nutrição e Metabolismo - Consumo Alimentar - Visualizando Porções.

[23] Previdelli AN, Andrade SC, Pires MM, Ferreira SR, Fisberg RM, Marchioni DM (2011). A revised version of the healthy eating index for the Brazilian population. Rev Saude Publica.

[24] Toffano RBD, Hillesheim E, Mathias MG, Coelho-Landell CA, Salomao RG, Almada M, et al. Validation of the Brazilian Healthy Eating Index-Revised Using Biomarkers in Children and Adolescents. Nutrients. 2018;10(2):154. 10.3390/nu10020154. 10.3390/nu10020154 PMC5852730 29385742

[25] Doyon A, Kracht D, Bayazit AK, Deveci M, Duzova A, Krmar RT (2013). Carotid artery intima-media thickness and distensibility in children and adolescents: reference values and role of body dimensions. Hypertension..

[26] Dalla Pozza R, Pirzer R, Beyerlein A, Weberruss H, Oberhoffer R, Schmidt-Trucksass A (2016). Beyond intima-media-thickness: analysis of the carotid intima-media-roughness in a paediatric population. Atherosclerosis..

[27] Jourdan C, Wuhl E, Litwin M, Fahr K, Trelewicz J, Jobs K (2005). Normative values for intima-media thickness and distensibility of large arteries in healthy adolescents. J Hypertens.

[28] Denarie N, Gariepy J, Chironi G, Massonneau M, Laskri F, Salomon J (2000). Distribution of ultrasonographically-assessed dimensions of common carotid arteries in healthy adults of both sexes. Atherosclerosis..

[29] Magnussen CG, Venn A, Thomson R, Juonala M, Srinivasan SR, Viikari JS (2009). The association of pediatric low- and high-density lipoprotein cholesterol dyslipidemia classifications and change in dyslipidemia status with carotid intima-media thickness in adulthood evidence from the cardiovascular risk in young Finns study, the Bogalusa heart study, and the CDAH (childhood determinants of adult health) study. J Am Coll Cardiol.

[30] Lattanzi B, Consolaro A, Solari N, Ruperto N, Martini A, Ravelli A (2011). Measures of disease activity and damage in pediatric systemic lupus erythematosus: British Isles Lupus Assessment Group (BILAG), European Consensus Lupus Activity Measurement (ECLAM), Systemic Lupus Activity Measure (SLAM), Systemic Lupus Erythematosus Disease Activity Index (SLEDAI), Physician's Global Assessment of Disease Activity (MD Global), and Systemic Lupus International Collaborating Clinics/American College of Rheumatology Damage Index (SLICC/ACR DI; SDI). Arthritis Care Res (Hoboken).

[31] Baragetti A, Ramirez GA, Magnoni M, Garlaschelli K, Grigore L, Berteotti M (2018). Disease trends over time and CD4(+)CCR5(+) T-cells expansion predict carotid atherosclerosis development in patients with systemic lupus erythematosus. Nutr Metab Cardiovasc Dis.

[32] Su-Angka N, Khositseth A, Vilaiyuk S, Tangnararatchakit K, Prangwatanagul W (2017). Carotid intima-media thickness and arterial stiffness in pediatric systemic lupus erythematosus. Lupus..

[33] Huang YL, Chung HT, Chang CJ, Yeh KW, Chen LC, Huang JL (2009). Lymphopenia is a risk factor in the progression of carotid intima-media thickness in juvenile-onset systemic lupus erythematosus. Arthritis Rheum.

[34] Strong JP, Malcom GT, Oalmann MC, Wissler RW (1997). The PDAY study: natural history, risk factors, and pathobiology. Pathobiological determinants of atherosclerosis in youth. Ann N Y Acad Sci.

[35] Kavey RE, Allada V, Daniels SR, Hayman LL, BW MC, Newburger JW (2006). Cardiovascular risk reduction in high-risk pediatric patients: a scientific statement from the American Heart Association Expert Panel on Population and Prevention Science; the Councils on Cardiovascular Disease in the Young, Epidemiology and Prevention, Nutrition, Physical Activity and Metabolism, High Blood Pressure Research, Cardiovascular Nursing, and the Kidney in Heart Disease; and the Interdisciplinary Working Group on Quality of Care and Outcomes Research: endorsed by the American Academy of Pediatrics. Circulation.

[36] Salomao RG, de Carvalho LM, Izumi C, Czernisz ES, Rosa JC, Antonini SRR (2018). Homocysteine, folate, hs-C-reactive protein, tumor necrosis factor alpha and inflammatory proteins: are these biomarkers related to nutritional status and cardiovascular risk in childhood-onset systemic lupus erythematosus?. Pediatr Rheumatol Online J.

[37] Nascif AK, Hilario MO, Terreri MT, Ajzen SA, D'Almeida V, Plavnik FL (2007). Endothelial function analysis and atherosclerotic risk factors in adolescents with systemic lupus erythematosus. Int J Adolesc Med Health.

[38] Abad TO, Sarni RO, da Silva SG, Machado D, Suano-Souza FI, Len CA (2018). Nutritional intervention in patients with juvenile systemic lupus erythematosus: protective effect against the increase in fat mass. Rheumatol Int.

[39] Barsalou J, Bradley TJ, Tyrrell PN, Slorach C, Ng LW, Levy DM (2016). Impact of disease duration on vascular surrogates of early atherosclerosis in childhood-onset systemic lupus Erythematosus. Arthritis Rheumatol.

[40] Lima DS, Sato EI, Lima VC, Miranda F, Hatta FH (2002). Brachial endothelial function is impaired in patients with systemic lupus erythematosus. J Rheumatol.

[41] Giannelou M, Mavragani CP (2017). Cardiovascular disease in systemic lupus erythematosus: a comprehensive update. J Autoimmun.

[42] Mende R, Vicent FB, Kandane-Rathnayake R, Koelmeyer R, Lin E, Chang J (2018). Analysis of serum interleukin (IL)-1beta and IL-18 in systemic lupus Erythematosus. Front Immunol.

[43] Ling S, Nheu L, Komesaroff PA (2012). Cell adhesion molecules as pharmaceutical target in atherosclerosis. Mini Rev Med Chem.

[44] Lewis MJ, Vyse S, Shields AM, Zou L, Khamashta M, Gordon PA (2016). Improved monitoring of clinical response in systemic lupus Erythematosus by longitudinal trend in soluble vascular cell adhesion molecule-1. Arthritis Res Ther.

[45] Da Rosa Franchi Santos LF, Stadtlober NP, Dall’Aqua LG, Scavuzzi BM, Guimarães PM, Flauzino T (2018). Increased adhesion molecule levels in systemic lupus erythematosus: relationships with severity of illness, autoimmunity, metabolic syndrome and cortisol levels. Lupus..

[46] Ammirati E, Moroni F, Magnoni M, Camici PG (2015). The role of T and B cells in human atherosclerosis and atherothrombosis. Clin Exp Immunol.

[47] Tumurkhuu G, Montano E, Jefferies C (2019). Innate immune Dysregulation in the development of cardiovascular disease in lupus. Curr Rheumatol Rep.

[48] Boros CA, Bradley TJ, Cheung MM, Bargman JM, Russell JL, McCrindle BW (2011). Early determinants of atherosclerosis in paediatric systemic lupus erythematosus. Clin Exp Rheumatol.

[49] Ajeganova S, Gustafsson T, Lindberg L, Hafstrom I, Frostegard J (2020). Similar progression of carotid intima-media thickness in 7-year surveillance of patients with mild SLE and controls, but this progression is still promoted by dyslipidaemia, lower HDL levels, hypertension, history of lupus nephritis and a higher prednisolone usage in patients. Lupus Sci Med.

[50] Sharma SK, Rathi M, Sahoo S, Prakash M, Dhir V, Singh S (2016). Assessment of premature atherosclerosis in systemic lupus erythematosus patients with and without nephritis. Lupus..

[51] Woo KS, Chook P, Yu CW, Sung RY, Qiao M, Leung SS (2004). Effects of diet and exercise on obesity-related vascular dysfunction in children. Circulation.

[52] Wiegman A, Hutten BA, de Groot E, Rodenburg J, Bakker HD, Büller HR (2004). Efficacy and safety of statin therapy in children with familial hypercholesterolemia: a randomized controlled trial. Jama.

